# Pathological mechanisms and crosstalk among various cell death pathways in cardiac involvement of systemic lupus erythematosus

**DOI:** 10.3389/fimmu.2024.1452678

**Published:** 2024-09-05

**Authors:** Jingjing Wei, Aolong Wang, Bin Li, Xingyuan Li, Rui Yu, Haitao Li, Xinlu Wang, Yongxia Wang, Mingjun Zhu

**Affiliations:** ^1^ Heart Center, The First Affiliated Hospital of Henan University of Chinese Medicine, Zhengzhou, China; ^2^ Henan Evidence-based Medicine Center of Chinese Medicine, The First Affiliated Hospital of Henan University of Chinese Medicine, Zhengzhou, China

**Keywords:** programmed cell death, systemic lupus erythematosus, cardiac involvement, crosstalk, immune dysregulation

## Abstract

Systemic lupus erythematosus (SLE) is a prevalent autoimmune disease primarily characterized by the involvement of multiple systems and organs. Cardiovascular disease is the primary cause of mortality in patients with SLE, though the mechanisms underlying the increased cardiovascular risk in SLE patients remain unclear. Recent studies indicate that abnormal activation of programmed cell death (PCD) signaling and the crosstalk among various forms of cell death are critical in the immunopathogenesis of SLE. Furthermore, apoptosis, necroptosis, pyroptosis, NETosis, and ferroptosis are recognized as key cellular processes in the pathogenesis of SLE and are closely linked to cardiac involvement. This review uniquely explores the intricate crosstalk between apoptosis, necroptosis, and other cell death pathways, discussing their roles and interactions in the pathogenesis of cardiac involvement in SLE. Investigating the interplay between PCD signaling and cardiac involvement in SLE in understanding the disease’s underlying mechanisms and offers opportunities for new therapeutic interventions. The integration of precision medicine and innovative strategies targeting these complex pathways holds promise for enhancing the treatment prospects of SLE with cardiac involvement.

## 1 Introduction

Systemic lupus erythematosus (SLE) is an autoimmune disease characterized by systemic inflammation and abnormal production of pathogenic antibodies (1). The clinical manifestations of SLE are highly heterogeneous, potentially affecting multiple organs including the heart, skin, skeletal muscles, kidneys, lungs, and gastrointestinal tract (2). Over the past 20 years, advances in medical technology have decreased the mortality rate of SLE patients, raising the 5-year survival rate to over 90% (3). However, the standardized mortality rate of SLE patients remains 2.5 times higher than that of the general population (4). Cardiovascular disease (CVD) is the leading cause of premature death in SLE patients (5), with the risk of CVD-related mortality being 3 times higher in SLE patients than in the general population (6). To further reduce the mortality rate of SLE patients, it is urgent to investigate the pathological mechanisms underlying cardiac involvement in systemic lupus erythematosus.

Multiple forms of programmed cell death (PCD) have been described in SLE patients and are considered contributing factors to disease onset (7). These cell death mechanisms not only activate the immune system through the release of autoantigens and inflammatory mediators but also influence the course and severity of SLE by regulating immune cell functions and inflammatory responses, ultimately causing damage to multiple organs (8). Previous studies have shown enhanced apoptosis in SLE patients with cardiac involvement (9). Additionally, necroptosis, pyroptosis, NETosis, and ferroptosis are known to be closely associated with CVD (10–12). Recent research indicates that the crosstalk between different forms of cell death has a significant impact on the pathogenesis of SLE and cardiac involvement. For instance, the interaction between apoptosis and necroptosis can provoke a stronger immune response and inflammatory reaction, worsening cardiovascular damage (13). Additionally, emerging forms of cell death, such as pyroptosis and ferroptosis, are increasingly studied in SLE, and their roles in cardiac involvement are attracting growing attention (14, 15). Some clinical trials investigating cell death mechanisms have further elucidated potential therapeutic targets for cardiac involvement in SLE (**Table 1**). A comprehensive delineation of different pathways mediating different cell death mechanisms and their interactions will help elucidate the pathological basis in cardiac involvement of SLE patients, providing a theoretical foundation for developing new therapeutic strategies.

**Table 1:** Overview of clinical studies on cell death mechanisms.

Classification	Condition or disease	ClinicalTrials.gov number	Sponsor	Interventions	Primary outcomes	Phase
IFN-1 receptor blocking antibody	Systemic lupus erythematosus, premature atherosclerosis	NCT05440422	National Institute of Arthritis and Musculoskeletal and Skin Diseases	Anifrolumab	Cardio-ankle vascular index, pulse wave velocity, vascular inflammation	Phase 2
	Active systemic lupus erythematosus	NCT02794285	AstraZeneca	Anifrolumab	Exposure-adjusted incidence rates of adverse events of special interest, serious adverse events	Phase 3
	Active systemic lupus erythematosus	NCT02446899	AstraZeneca	Anifrolumab	Number of participants who achieved British Isles Lupus Assessment Group-based Composite Lupus Assessment	Phase 3
	Active systemic lupus erythematosus	NCT02446912	AstraZeneca	Anifrolumab	Number of participants who achieved a systemic lupus erythematosus responder index ≥4	Phase 3
	Systemic lupus erythematosus	NCT04877691	AstraZeneca	Anifrolumab	British Isles Lupus Assessment Group-based Composite Lupus Assessment response	Phase 3
NLRP3 inflammasome inhibitor	Healthy subjects	NCT04015076	Inflazome UK Ltd	Inzomelid	Incidence of treatment emergent advert events, peak plasma concentration	Phase 1
MPO inhibitor	healthy male subjects	NCT04986202	AstraZeneca	AZD4831	Frequencies of adverse events	Phase 1
	Healthy subjects	NCT04232345	AstraZeneca	AZD4831	Number of subjects with adverse events/serious adverse events	Phase 1
	Heart failure	NCT03756285	AstraZeneca	AZD4831	MPO specific activity	Phase 2
	Heart failure	NCT03611153	Mayo clinic	AZD4831	Exercise Pulmonary capillary wedge pressure	Phase 1 and Phase 2
	Heart failure with preserved ejection fraction	NCT04986202	AstraZeneca	AZD4831	Kansas city cardiomyopathy questionnaire–total symptom score, six minute walk distance	Phase 2b and Phase 3

## 2 Apoptosis and its contribution to cardiac involvement

**Figure 1:**
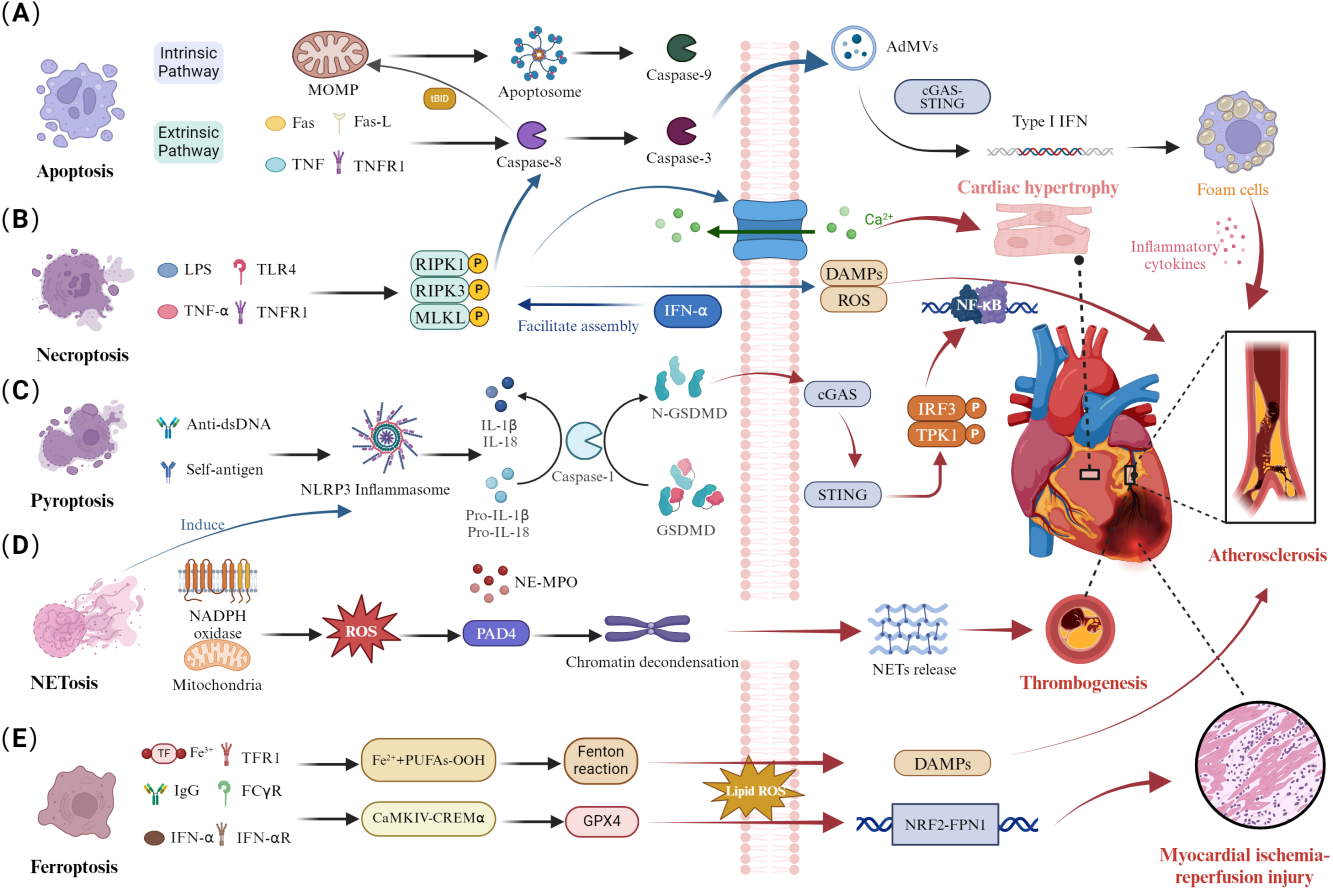
Dysregulation of cell death pathways and cardiac involvement in SLE. **(A)** Disruption of apoptosis. The extrinsic apoptotic pathway activates caspase-8 through specific receptor-ligand interactions, converting it to caspase-3. Additionally, caspase-8 cleaves Bid, inducing mitochondrial outer MOMP and activating caspase-9 within the apoptosome, triggering a caspase cascade. Activation of caspase-3 generates AdMVs, which induce IFN-1 production via the cGAS-STING pathway, influencing atherosclerotic plaque progression by enhancing foam cell formation. **(B)** Necroptosis disturbance. Activation of necroptosis through TNFR and TLR4 binding leads to caspase-8-mediated apoptosis. The necrosome is stabilized by RIPK1, RIPK3, and autophosphorylated MLKL, which promotes the binding of phosphorylated MLKL to the cell membrane. This process disrupts cell integrity, exposing DAMPs. The RIPK3/MLKL signaling pathway mediates calcium influx, promoting cardiomyocyte hypertrophy. **(C)** Pyroptotic imbalance. Anti-dsDNA antibodies and autoantigens activate the NLRP3 inflammasome, leading to caspase-1-mediated maturation and secretion of IL-1β and IL-18, along with GSDMD cleavage. Subsequently, GSDMD can mediate the cGAS/STING/TBK1/IRF3/NF-κB signaling pathway, contributing to the atherosclerosis process. **(D)** Abnormal NETosis. NADPH oxidase produces ROS and mitochondrial ROS, activating PAD4 and inducing chromatin decondensation. The nuclear translocation of NE and MPO leads to histone degradation and NETs release, contributing to arterial thrombosis formation. **(E)** Ferroptosis dysregulation. Autoantibodies and IFN-α synergistically induce the CaMKIV/CREMα axis, inhibiting GPX4 expression and increasing lipid-derived ROS, exacerbating ferroptosis. The NRF2/FPN1 signaling pathway is downregulated, leading to myocardial ischemia-reperfusion injury.

Apoptosis is a form of programmed cell death that is activated via two pathways: the extrinsic pathway, which activates caspase-8 through specific receptor-ligand interactions, and the intrinsic pathway, which activates caspase-9 through the mitochondrial pathway, ultimately leading to apoptosis by activating caspase-3 (**Figure 1A**) (16). Additionally, the caspase-8 mediated apoptotic pathway can drive mitochondrial outer membrane permeabilization (MOMP) by cleaving BID, thereby activating caspase-9 and caspase-3 (17). The activation of caspase-3 leads to the production of apoptosis-derived membrane vesicles (AdMVs) containing double-stranded DNA (dsDNA). It is noteworthy that extracellular vesicles can be released during cellular death processes, including necroptosis, pyroptosis, and NETosis. The defective clearance of extracellular vesicles resulting from excessive cell death can trigger autoimmune responses, which is one of the pathological mechanisms of systemic lupus erythematosus (18, 19). The cyclic GMP-AMP synthase-stimulator of interferon genes (cGAS-STING) signaling pathway is the primary effector by which cells sense and respond to the abnormal presence of dsDNA in the cytoplasm (20). In the serum of SLE patients, AdMVs induce type I interferon (IFN-1) production through the cGAS-STIN pathway (21). Notably, senescent monocytes in SLE can also promote IFN-α production via the STING pathway (22). IFN-1 is a key cytokine mediator in the pathogenesis of SLE, with elevated levels of interferon-stimulated genes in the blood of systemic lupus erythematosus patients (23). IFN-1 can mediate CD8+ T cell death in SLE patients by regulating nicotinamide adenine dinucleotide (NAD+) levels (24). Additionally, recent studies have confirmed that IFNα is associated with B cell activation and autoantibody production (25, 26). In summary, Apoptosis is closely related to SLE. Increased apoptosis and defective clearance of apoptotic cells lead to heightened autoantigen exposure, triggering autoimmune and inflammatory responses in SLE (7). This may be one of the reasons for the involvement of multiple organs.

Previous studies indicated that patients with SLE have a significantly increased risk of CV (27, 28). Patients with both SLE and CVD exhibited higher levels of fas cell surface death receptor (FAS), tumor necrosis factor receptor 1(TNFR1), matrix metallopeptidase 1 (MMP-1), and MMP-7 compared to those without CVD (9). This suggested that FAS and TNFR1-mediated extrinsic apoptosis may be linked to the heightened cardiovascular risk in SLE patients. A clinical trial involving 80 SLE patients revealed that anti-dsDNA antibodies can promote monocyte apoptosis and activate the immune system, thereby driving the development of atherosclerosis (29). Additionally, studies have indicated that the mitochondrial deoxyribonucleic acid (mtDNA)-mediated cGAS-STING pathway is associated with endothelial-to-mesenchymal transition (30). Furthermore, apoptosis-mediated IFN-I may be a key factor in the cardiac involvement observed in SLE patients (31, 32). IFN-I induces apoptosis in endothelial progenitor cells and circulating angiogenic cells (33), promotes foam cell formation, activates endothelial and immune cells, and enhances the recruitment of pro-inflammatory leukocytes to the arteries (34, 35). This is a primary mechanism of atherosclerosis in SLE. A clinical trial indicated that Anifrolumab, an antibody blocking the IFN-I receptor, can reduce levels of neutrophil extracellular traps (NETs), TNF-α, and interleukin-10 (IL-10) while improving cholesterol efflux capacity (36). Overall, apoptosis and IFN-I generation may accelerate the progression of CVD in SLE. This mechanism could be a potential therapeutic target for reducing cardiovascular risk in SLE patients.

## 3 Necroptosis and its contribution to cardiac involvement

As we delve deeper into the specific mechanisms of apoptosis in SLE, it is essential to understand how this process interacts with other cell death pathways, such as necroptosis. Necroptosis is a form of cell death distinct from traditional apoptosis and necrosis pathways. This process is mediated by receptor-interacting protein 1 (RIPK1), RIPK3, and mixed lineage kinase domain-like protein (MLKL) (**Figure 1B**) (37). The formation of the RIPK1/RIPK3 necrosome induces MLKL phosphorylation, which plays a role in the inflammation and immune response in SLE (38, 39). The crosstalk between apoptosis, necroptosis, and pyroptosis has emerged as a crucial pathological mechanism in SLE. The inhibition of caspase-8 activity in apoptosis can activate RIPK3 and MLKL phosphorylation, leading to necroptosis (16). Enhanced INF signaling in SLE patients can mediate the assembly of RIPK1 and RIPK3, increasing necroptosis levels (40). Interestingly, RIPK3 can mediate both apoptosis and necroptosis, depending on the levels of its molecular chaperones, heat shock protein 90 (Hsp90), and cell division cycle protein 37 (Cdc37). RIPK3 can aggregate with RIPK1, and caspase-8 to activate apoptosis (41). Additionally, the activation of RIPK3 and MLKL can promote the production of nucleotide-binding oligomerization domain-like receptor protein 3 (NLRP3) and IL-1β, which are associated with pyroptosis (42). These findings indicate that necroptosis mediates the processes of apoptosis and pyroptosis.

MLKL, a critical factor in necroptosis, has increased mRNA levels in the peripheral blood mononuclear cells (PBMCs) of SLE patients (43). Meanwhile, the RIPK1/RIPK3/MLKL pathway-mediated necroptosis plays a significant role in various CVDs, including atherosclerosis, ischemia-reperfusion injury, myocardial infarction, and myocarditis (44, 45). We hypothesize that necroptosis might be a contributing factor to cardiac involvement in SLE. In patients with atherosclerosis, the expression levels of RIPK3 protein and MLKL are elevated. The necroptosis inhibitor Necrostatin-1 can reduce necrosome formation, thereby alleviating plaque instability and size (46). Additionally, studies indicate that RIPK1 may contribute to atherosclerosis by activating the NF-κB pathway and promoting inflammation (47). Interestingly, research also suggests that RIPK1 is involved in endothelial cell damage induced by oxidized low-density lipoprotein (ox-LDL), and the necroptosis inhibitor Necrostatin-1 can inhibit the NF-κB pathway (48). RIPK3 protein expression increases in models of cardiomyocyte hypertrophy, and the RIPK3/MLKL signaling pathway can mediate calcium influx, promoting cardiomyocyte hypertrophy (49). In mouse models, the RIPK3 inhibitor GSK872 can ameliorate diabetes-induced myocardial fibrosis (50). These studies elucidate the importance of RIPK1/RIPK3/MLKL pathway-mediated necroptosis in SLE and CVD. Despite the current lack of research on the necroptosis mechanisms in both conditions, targeting necroptosis might be a potential strategy for reducing CVD risk in SLE. In addition to apoptosis and necroptosis, pyroptosis is also an important factor in the pathological mechanism of cardiac involvement in SLE.

## 4 Pyroptosis and its contribution to cardiac involvement

Pyroptosis is a novel form of PCD characterized by membrane perforation, cell rupture, and release of inflammatory cytokines, including IL-1β and IL-18, mediated by the gasdermin D (GSDMD) protein family triggered by inflammasomes, promoting inflammatory responses and rapidly initiating the immune response process (**Figure 1C**) (51). NLRP3 can activate caspase-1, which cleaves IL-1β and IL-18 precursors into mature inflammatory factors IL-1β and IL-18, further exacerbating the occurrence of inflammatory responses and inducing pyroptosis (52). Moreover, GSDMD also plays a crucial role in SLE, where it is cleaved by caspase-1 into GSDMD-N, which translocates to the cell membrane and forms pores, facilitating the release of IL-1β and IL-18 (53). Importantly, abnormal aggregation or clearance defects of apoptotic cells in SLE patients can lead to exposure of self-antigens, accumulation of DNA and mtDNA, promoting the production of autoantibodies, and further enhancing the generation of IFN-1 (54, 55). Recent research using a SLE mouse model has confirmed that mtDNA promotes the oligomerization of GSDMD. The GSDMD inhibitor DSF can reduce the production of autoantibodies, suggesting GSDMD as a new therapeutic target for SLE (56). Additionally, DSF, as a GSDMD inhibitor, effectively suppresses the levels of IFN and pro-inflammatory cytokines in the PBMC of SLE patients.

The GSDMD, as a key mediator of cell pyroptosis, is closely associated not only with pathological process in SLE but also with the inflammatory response during acute myocardial infarction. Inhibition of GSDMD can reduce the area of myocardial infarction, exerting a cardioprotective function (57). Recent research has shown enhanced expression of GSDMD in atherosclerosis, where it can mediate the cGAS/STING/TBK1/IRF3/NF-κB signaling pathway, contributing to the process of atherosclerosis. GSDMD inhibitors can reduce the formation of atherosclerotic plaques by regulating the release of mtDNA and the STING pathway (58). Furthermore, PBMCs in SLE patients exhibit upregulated expression of NLRP3 and IL-1β (59). Previous studies have reported the involvement of NLRP3 inflammasomes and pro-inflammatory cytokines IL-1β and IL-18 in the development of various CVDs (10, 60), including acute myocardial infarction, atherosclerosis, and heart failure. Elevated levels of NLRP3 inflammasomes, caspase-1, and IL-1β are observed in the peripheral blood of patients with dilated cardiomyopathy, with NLRP3 levels serving as an independent risk factor for readmission within 6 months (61). The NLRP3 inhibitor MCC950 may represent a potential therapeutic avenue for ameliorating multi-organ involvement in patients with SLE (62). Recent research has confirmed that the MCC950 can alleviate oxidative stress and aging in myocardial cells, alleviate adrenaline-induced cardiac dysfunction in mice, and reduce the formation of atherosclerotic plaques and systemic inflammation (63, 64). Mounting evidence suggests that IL-1 and NLRP3 inflammasomes may serve as novel therapeutic targets for CVDs. Previous research has indicated that pyroptosis mediated by GSDMD and NLRP3 plays similar roles in both SLE and CVDs (65, 66). Moreover, GSDMD inhibitors exhibit significant efficacy in treating both SLE and CVDs, possibly offering a direction for future treatments of these conditions. In addition to the above-mentioned modes of cell death, NETosis also plays an important role in cardiac involvement in SLE.

## 5 NETosis and its contribution to cardiac involvement

NETs are web-like structures released by neutrophils into the extracellular space (67). The formation of NETs is accompanied by neutrophil death, termed NETosis, which is a unique cell death process distinct from apoptosis and necroptosis. It is known that the formation of NETosis is closely related to reactive oxygen species (ROS) produced by NADPH oxidase and mitochondria (**Figure 1D**). Activated peptidyl arginine deiminase 4 (PAD4) mediates histone citrullination and neutrophil elastase (NE)-mediated histone cleavage, facilitating chromatin decondensation, a critical process in the formation of Neutrophil Extracellular Traps (NETs) (68). Furthermore, the rupture of the nuclear and plasma membranes is essential for the extracellular release of chromatin and NET formation (69). The nuclear membrane acts as the primary physical barrier to chromatin release, while the nuclear lamina, composed of type A (A, C) and type B (B1, B2) lamin filaments, plays a critical role in maintaining its structural integrity (70). Recent research has revealed that the rupture of the nuclear membrane during NET formation is caused by kinase-mediated phosphorylation and disassembly of the nuclear lamina, rather than by proteolytic cleavage (71). For instance, cyclin-dependent kinases 4 and 6(CDK4/6), associated with nuclear envelope rupture, may regulate the phosphorylation of lamin A/C, although causality has not been experimentally confirmed (72). Notably, protein kinase C-α (PKC-α) plays a pivotal role in the phosphorylation of lamin B, facilitating the nuclear envelope rupture during NETosis (71). Recent studies have confirmed that NETs are an important source of mononucleosome circulating DNA, distinct from apoptotic cells (73). The DNA, granule proteins, and histones released during NETosis can become autoantigens in SLE (73). Furthermore, the formation of NETs can promote IFN secretion, accelerating inflammation and disease progression in SLE (74). Simultaneously, apoptotic cell microparticles in SLE patients drive the formation of NETosis (75, 76). Notably, GSDMD plays a significant role in pyroptosis in SLE. Recent studies have shown that GSDMD knockout inhibits NETs formation in SLE mouse models (56). Additionally, mitochondrial ROS participate in both NETs formation and IFN-1 production. Inhibition of mitochondrial ROS reduces IFN-1 production, alleviating disease progression in SLE (77, 78). These studies illustrate the crosstalk between pyroptosis, NETs, and apoptosis in SLE, ultimately leading to immune system dysregulation in SLE.

Recent studies have found elevated levels of NETs in the circulating blood of SLE patients (79). It is known that NETs are involved in processes closely related to CVDs, such as atherosclerosis (12), thrombosis (80), and adverse cardiac remodeling (81). Stanley Moore and colleagues conducted the first clinical study on the correlation between CVD and NETs in SLE patients. The results showed that NETs are related to CVDs such as arterial thrombosis and endothelial cell activation (82). The active components of NETs, such as DNA and histones, can regulate the levels of FXII and FXI (83, 84). The crosstalk between NETs and endothelial cell mechanisms jointly participates in thrombosis formation (85). Additionally, NETs can be elevated in patients with primary hypertension, but their levels decrease after ARB treatment. This phenomenon may be related to oxidative stress, autophagy, and PAD4-mediated histone citrullination induced by Ang II (86). A recent mouse study confirmed that Padi4-/- mice have a weakened hypertensive response to Ang II, reduced aortic inflammation, and improved endothelial cell-dependent vasorelaxation (87). Furthermore, PAD inhibition can alleviate atherosclerosis and arterial thrombosis formation (88). On the other hand, MPO contributes to CVD by promoting endothelial dysfunction, activating MMP, and causing LDL and HDL imbalances. MPO is associated with atherosclerotic plaques and adverse cardiovascular events (89). MPO inhibitors (AZM198) can stabilize plaques, and serum MPO-DNA complexes can serve as biomarkers for predicting adverse left ventricular remodeling post-PCI in myocardial infarction patients (90, 91). In summary, although MPO, PAD4, and NETs formation are related to CVD, extensive clinical trials and basic research are still ongoing. This suggests that NETs may become a common target for future treatment of SLE and CVD. As we consider the role of NETosis in SLE, it is crucial to also examine ferroptosis. The interaction between NETosis and ferroptosis adds another dimension to the complex network of cell death modalities in SLE.

## 6 Ferroptosis and its contribution to cardiac involvement

Ferroptosis is a form of non-apoptotic cell death characterized by abnormal iron metabolism, lipid metabolic disorders, and excessive accumulation of ROS (**Figure 1E**) (92). The glutathione (GSH)-glutathione peroxidase 4 (GPX4) axis and the accumulation of iron ions, which trigger the Fenton reaction, mediate lipid peroxidation, ultimately leading to ferroptosis (93). GPX4 is a key regulator in the ferroptosis process, capable of decomposing lipid peroxides (LPO), thereby inhibiting ferroptosis (94). Previous studies have shown that serum autoantibodies and IFN-α in SLE patients can regulate the CaMKIV/CREMα signaling pathway, inducing neutrophil ferroptosis by inhibiting GPX4 activity and leading to increased lipid ROS (95). Ferroptosis may be a key process in neutrophil death and reduction in SLE patients. Recent studies have confirmed that B cell ferroptosis is involved in the progression of SLE, potentially related to ferroptosis-mediated B cell differentiation and plasma cell composition. Ferroptosis inhibitors, such as Liproxstatin-1, inhibited the production of autoantibodies and malondialdehyde in SLE (96, 97). These studies highlight the significant role of ferroptosis in different cells during the progression of SLE.

Interestingly, ferroptosis is widely involved in the development of various CVDs, including atherosclerosis, myocardial infarction, ischemia-reperfusion injury, and heart failure (98). It is known that ox-LDL can induce endothelial dysfunction associated with atherosclerosis (99). Iron overload promotes macrophages to absorb ox-LDL and transform into foam cells, ultimately leading to atherosclerosis. In ox-LDL-treated mouse aortic endothelial cells, the ferroptosis inhibitor Ferrostatin-1 can regulate lipid peroxidation and ferroptosis. Ferrostatin-1 alleviates endothelial dysfunction and atherosclerosis by downregulating adhesion molecules and upregulating eNOS expression (100). Meanwhile, GPX4 plays a key role in ferroptosis. Previous studies have shown that the severity of atherosclerosis is negatively correlated with GPX4 expression (101). In I/R mouse models, GPX4 expression is significantly reduced, which may be related to the upregulation of ELAVL1 and the NRF2/FPN1 pathway (102, 103). Ferroptosis inhibitors, such as Liproxstatin-1, can protect the heart by regulating GPX4 expression (104). Although there is currently a lack of research on the shared mechanisms of ferroptosis in SLE and CVD, existing studies suggest that the ferroptosis process in SLE is closely related to CVD. This understanding may help us better explore the reasons for the higher risk of CVD in SLE patients.

## 7 Conclusion

Overall, increasing research provides compelling evidence for the involvement of various cell death modalities in cardiac complications associated with SLE, highlighting a higher risk of cardiovascular disease-related mortality in SLE patients. There is an urgent need to understand the common mechanisms between SLE and CVD to develop new therapeutic strategies. In this review, we systematically discuss the potential common mechanisms linking different types of cell death and immune response crosstalk in SLE patients and their contribution to cardiac involvement, revealing potential common targets. This may represent just the tip of the iceberg of SLE-related cardiac complications. Although numerous studies have shown that the crosstalk between different cell death modalities leads to autoimmune abnormalities in SLE, which may underlie the increased cardiovascular risk, more basic and clinical research is needed to confirm this. Understanding the crosstalk between cell death pathways not only elucidates the pathogenesis of cardiac involvement in SLE but also opens new avenues for therapeutic intervention. By comprehensively mapping these interactions, we can better identify novel targets for treatment, potentially improving outcomes for patients with SLE and reducing their cardiovascular risk. Future research should focus on elucidating these mechanisms further, with the aim of translating these findings into clinical applications.

## References

[1] Kiriakidou M Ching CL. Systemic lupus erythematosus. Ann Intern Med. (2020) 172:ITC81–96. doi: 10.7326/AITC202006020 32479157

[2] Dörner T Furie R. Novel paradigms in systemic lupus erythematosus. Lancet. (2019) 393:2344–58. doi: 10.1016/S0140-6736(19)30546-X 31180031

[3] Barber MRW Drenkard C Falasinnu T Hoi A Mak A Kow NY. Global epidemiology of systemic lupus erythematosus. Nat Rev Rheumatol. (2021) 17:515–32. doi: 10.1038/s41584-021-00668-1 PMC8982275 34345022

[4] Barber MRW Falasinnu T Ramsey-Goldman R Clarke AE. The global epidemiology of SLE: narrowing the knowledge gaps. Rheumatol (Oxford). (2023) 62:i4–9. doi: 10.1093/rheumatology/keac610 PMC10050933 36987602

[5] Manadan AM Kambhatla S Gauto-Mariotti E Okoli C Block JA. Reasons for hospitalization and in-hospital mortality in adult systemic lupus erythematosus. ACR Open Rheumatol. (2020) 2:683–9. doi: 10.1002/acr2.11195 PMC7672299 33164350

[6] Taylor T Anastasiou C Ja C Rush S Trupin L Dall'Era M. Causes of death among individuals with systemic lupus erythematosus by race and ethnicity: A population-based study. Arthritis Care Res (Hoboken). (2023) 75:61–8. doi: 10.1002/acr.24988 PMC9797422 35904969

[7] Lou H Ling GS Cao X. Autoantibodies in systemic lupus erythematosus: From immunopathology to therapeutic target. J Autoimmun. (2022) 132:102861. doi: 10.1016/j.jaut.2022.102861 35872103

[8] Xu Y Li P Li K Li N Liu H Zhang X. Pathological mechanisms and crosstalk among different forms of cell death in systemic lupus erythematosus. J Autoimmun. (2022) 132:102890. doi: 10.1016/j.jaut.2022.102890 35963809

[9] Wigren M Svenungsson E Mattisson IY Gustafsson JT Gunnarsson I Zickert A. Cardiovascular disease in systemic lupus erythematosus is associated with increased levels of biomarkers reflecting receptor-activated apoptosis. Atherosclerosis. (2018) 270:1–7. doi: 10.1016/j.atherosclerosis.2018.01.022 29407876

[10] Toldo S Abbate A. The role of the NLRP3 inflammasome and pyroptosis in cardiovascular diseases. Nat Rev Cardiol. (2024) 21:219–37. doi: 10.1038/s41569-023-00946-3 PMC11550901 37923829

[11] Zhang ZD Shi CR Li FX Gan H Wei Y Zhang Q. Disulfiram ameliorates STING/MITA-dependent inflammation and autoimmunity by targeting RNF115. Cell Mol Immunol. (2024) 21:275–91. doi: 10.1038/s41423-024-01131-3 PMC10901794 38267694

[12] Liu Y Carmona-Rivera C Moore E Seto NL Knight JS Pryor M. Myeloid-specific deletion of peptidylarginine deiminase 4 mitigates atherosclerosis. Front Immunol. (2018) 9:1680. doi: 10.3389/fimmu.2018.01680 30140264 PMC6094966

[13] Frostegård J. Systemic lupus erythematosus and cardiovascular disease. J Intern Med. (2023) 293:48–62. doi: 10.1111/joim.13557 35982610 PMC10087345

[14] Bello N Meyers KJ Workman J Hartley L McMahon M. Cardiovascular events and risk in patients with systemic lupus erythematosus: Systematic literature review and meta-analysis. Lupus. (2023) 32:325–41. doi: 10.1177/09612033221147471 PMC10012401 36547368

[15] Katz G Smilowitz NR Blazer A Clancy R Buyon JP Berger JS. Systemic lupus erythematosus and increased prevalence of atherosclerotic cardiovascular disease in hospitalized patients. Mayo Clin Proc. (2019) 94:1436–43. doi: 10.1016/j.mayocp.2019.01.044 PMC6711365 31303426

[16] Ai Y Meng Y Yan B Zhou Q Wang X. The biochemical pathways of apoptotic, necroptotic, pyroptotic, and ferroptotic cell death. Mol Cell. (2024) 84:170–9. doi: 10.1016/j.molcel.2023.11.040 38181758

[17] Tan Q Huang W Zheng Y Li M Tao Y Yu S. Unveiling the nexus: decoding interactions between regulated cell death and systemic lupus erythematosus pathogenesis for innovative therapeutic avenues. Rheumatol Autoimmun. (2024) 4:1–10. doi: 10.1002/rai2.12104

[18] Zhao Y Wei W Liu ML. Extracellular vesicles and lupus nephritis - New insights into pathophysiology and clinical implications. J Autoimmun. (2020) 115:102540. doi: 10.1016/j.jaut.2020.102540 32893081 PMC9107953

[19] Liu ML Williams KJ Werth VP. Microvesicles in autoimmune diseases. Adv Clin Chem. (2016) 77:125–75. doi: 10.1016/bs.acc.2016.06.005 27717416

[20] Chen C Xu P. Cellular functions of cGAS-STING signaling. Trends Cell Biol. (2023) 33:630–48. doi: 10.1016/j.tcb.2022.11.001 36437149

[21] Kato Y Park J Takamatsu H Aoki W Aburaya S Ueda M. Apoptosis-derived membrane vesicles drive the cGAS-STING pathway and enhance type I IFN production in systemic lupus erythematosus. Ann Rheum Dis. (2018) 77:1507–15. doi: 10.1136/annrheumdis-2018-212988 PMC6161667 29945921

[22] Kuga T Chiba A Murayama G Hosomi K Nakagawa T Yahagi Y. Enhanced GATA4 expression in senescent systemic lupus erythematosus monocytes promotes high levels of IFNα production. Front Immunol. (2024) 15:1320444. doi: 10.3389/fimmu.2024.1320444 38605949 PMC11007064

[23] Caielli S Wan Z Pascual V. Systemic lupus erythematosus pathogenesis: interferon and beyond. Annu Rev Immunol. (2023) 41:533–60. doi: 10.1146/annurev-immunol-101921-042422 36854182

[24] Buang N Tapeng L Gray V Sardini A Whilding C Lightstone L. Type I interferons affect the metabolic fitness of CD8+ T cells from patients with systemic lupus erythematosus. Nat Commun. (2021) 12:1980. doi: 10.1038/s41467-021-22312-y 33790300 PMC8012390

[25] Ferri DM Nassar C Manion KP Kim M Baglaenko Y Muñoz-Grajales C. Elevated levels of interferon-α Act directly on B cells to breach multiple tolerance mechanisms promoting autoantibody production. Arthritis Rheumatol. (2023) 75:1542–55. doi: 10.1002/art.42482 36807718

[26] Baker T Sharifian H Newcombe PJ Gavin PG Lazarus MN Ramaswamy M. Type I interferon blockade with anifrolumab in patients with systemic lupus erythematosus modulates key immunopathological pathways in a gene expression and proteomic analysis of two phase 3 trials. Ann Rheum Dis. (2024) 83:1018–27. doi: 10.1136/ard-2023-225445 PMC12056589 38569851

[27] Lu X Wang Y Zhang J Pu D Hu N Luo J. Patients with systemic lupus erythematosus face a high risk of cardiovascular disease: A systematic review and Meta-analysis. Int Immunopharmacol. (2021) 94:107466. doi: 10.1016/j.intimp.2021.107466 33636561

[28] Kain J Owen KA Marion MC Langefeld CD Grammer AC Lipsky PE. Mendelian randomization and pathway analysis demonstrate shared genetic associations between lupus and coronary artery disease. Cell Rep Med. (2022) 3:100805. doi: 10.1016/j.xcrm.2022.100805 36334592 PMC9729823

[29] Patiño-Trives AM Pérez-Sánchez C Pérez-Sánchez L Luque-Tévar M Ábalos-Aguilera MC Alcaide-Ruggiero L. Anti-dsDNA antibodies increase the cardiovascular risk in systemic lupus erythematosus promoting a distinctive immune and vascular activation. Arterioscler Thromb Vasc Biol. (2021) 41:2417–30. doi: 10.1161/ATVBAHA.121.315928 34320837

[30] Liu Q Cheng Z Huang B Luo S Guo Y. Palmitic acid promotes endothelial-to-mesenchymal transition via activation of the cytosolic DNA-sensing cGAS-STING pathway. Arch Biochem Biophys. (2022) 727:109321. doi: 10.1016/j.abb.2022.109321 35697075

[31] Ambler WG Kaplan MJ. Vascular damage in systemic lupus erythematosus. Nat Rev Nephrol. (2024) 20:251–65. doi: 10.1038/s41581-023-00797-8 PMC11391830 38172627

[32] Ding X Xiang W He X. IFN-I mediates dysfunction of endothelial progenitor cells in atherosclerosis of systemic lupus erythematosus. Front Immunol. (2020) 11:581385. doi: 10.3389/fimmu.2020.581385 33262760 PMC7686511

[33] Blachut D Przywara-Chowaniec B Tomasik A Kukulski T Morawiec B. Update of potential biomarkers in risk prediction and monitoring of atherosclerosis in systemic lupus erythematosus to prevent cardiovascular disease. Biomedicines. (2023) 11:2814. doi: 10.3390/biomedicines11102814 37893187 PMC10604001

[34] Yennemadi AS Jordan N Diong S Keane J Leisching G. The link between dysregulated immunometabolism and vascular damage: implications for the development of atherosclerosis in systemic lupus erythematosus and other rheumatic diseases. J Rheumatol. (2024) 51:234–41. doi: 10.3899/jrheum.2023-0833 38224981

[35] Liu Y Yu X Zhang W Zhang X Wang M Ji F. Mechanistic insight into premature atherosclerosis and cardiovascular complications in systemic lupus erythematosus. J Autoimmun. (2022) 132:102863. doi: 10.1016/j.jaut.2022.102863 35853760

[36] Casey KA Smith MA Sinibaldi D Seto NL Playford MP Wang X. Modulation of cardiometabolic disease markers by type I interferon inhibition in systemic lupus erythematosus. Arthritis Rheumatol. (2021) 73:459–71. doi: 10.1002/art.41518 PMC11302498 32909675

[37] Dhuriya YK Sharma D. Necroptosis: a regulated inflammatory mode of cell death. J Neuroinflamm. (2018) 15:199. doi: 10.1186/s12974-018-1235-0 PMC6035417 29980212

[38] Wang H Sun L Su L Rizo J Liu L Wang LF. Mixed lineage kinase domain-like protein MLKL causes necrotic membrane disruption upon phosphorylation by RIP3. Mol Cell. (2014) 54:133–46. doi: 10.1016/j.molcel.2014.03.003 24703947

[39] Guo C Fu R Zhou M Wang S Huang Y Hu H. Pathogenesis of lupus nephritis: RIP3 dependent necroptosis and NLRP3 inflammasome activation. J Autoimmun. (2019) 103:102286. doi: 10.1016/j.jaut.2019.05.014 31133359 PMC6708470

[40] Sarhan J Liu BC Muendlein HI Weindel CG Smirnova I Tang AY. Constitutive interferon signaling maintains critical threshold of MLKL expression to license necroptosis. Cell Death Differ. (2019) 26:332–47. doi: 10.1038/s41418-018-0122-7 PMC6329789 29786074

[41] Li D Chen J Guo J Li L Cai G Chen S. A phosphorylation of RIPK3 kinase initiates an intracellular apoptotic pathway that promotes prostaglandin2α-induced corpus luteum regression. Elife. (2021) 10:e67409. doi: 10.7554/eLife.67409 34029184 PMC8143796

[42] Conos SA Chen KW De Nardo D Hara H Whitehead L Núñez G. Active MLKL triggers the NLRP3 inflammasome in a cell-intrinsic manner. Proc Natl Acad Sci USA. (2017) 114:E961–9. doi: 10.1073/pnas.1613305114 PMC5307433 28096356

[43] Zhang M Jie H Wu Y Han X Li X He Y. Increased MLKL mRNA level in the PBMCs is correlated with autoantibody production, renal involvement, and SLE disease activity. Arthritis Res Ther. (2020) 22:239. doi: 10.1186/s13075-020-02332-7 33054864 PMC7557011

[44] Zhe-Wei S Li-Sha G Yue-Chun L. The role of necroptosis in cardiovascular disease. Front Pharmacol. (2018) 9:721. doi: 10.3389/fphar.2018.00721 30034339 PMC6043645

[45] Zhao H Jaffer T Eguchi S Wang Z Linkermann A Ma D. Role of necroptosis in the pathogenesis of solid organ injury. Cell Death Dis. (2015) 6:e1975. doi: 10.1038/cddis.2015.316 26583318 PMC4670925

[46] Karunakaran D Geoffrion M Wei L Gan W Richards L Shangari P. Targeting macrophage necroptosis for therapeutic and diagnostic interventions in atherosclerosis. Sci Adv. (2016) 2:e1600224. doi: 10.1126/sciadv.1600224 27532042 PMC4985228

[47] Karunakaran D Nguyen MA Geoffrion M Vreeken D Lister Z Cheng HS. RIPK1 expression associates with inflammation in early atherosclerosis in humans and can be therapeutically silenced to reduce NF-κB activation and atherogenesis in mice. Circulation. (2021) 143:163–77. doi: 10.1161/CIRCULATIONAHA.118.038379 33222501

[48] An S Qi Y Zhang Z Mo R Hou L Yao X. Antagonism of receptor interacting protein 1 using necrostatin-1 in oxidized LDL- induced endothelial injury. BioMed Pharmacother. (2018) 108:1809–15. doi: 10.1016/j.biopha.2018.09.052 30372886

[49] Xue H Shi H Zhang F Li H Li C Han Q. RIP3 contributes to cardiac hypertrophy by influencing MLKL-mediated calcium influx. Oxid Med Cell Longev. (2022) 2022:5490553. doi: 10.1155/2022/5490553 35464769 PMC9023175

[50] Qiao S Hong L Zhu Y Zha J Wang A Qiu J. RIPK1-RIPK3 mediates myocardial fibrosis in type 2 diabetes mellitus by impairing autophagic flux of cardiac fibroblasts. Cell Death Dis. (2022) 13:147. doi: 10.1038/s41419-022-04587-1 35165268 PMC8844355

[51] You R He X Zeng Z Zhan Y Xiao Y Xiao R. Pyroptosis and its role in autoimmune disease: A potential therapeutic target. Front Immunol. (2022) 13:841732. doi: 10.3389/fimmu.2022.841732 35693810 PMC9174462

[52] Ren W Sun Y Zhao L Shi X. NLRP3 inflammasome and its role in autoimmune diseases: A promising therapeutic target. BioMed Pharmacother. (2024) 175:116679. doi: 10.1016/j.biopha.2024.116679 38701567

[53] Liu X Zhang Z Ruan J Pan Y Magupalli VG Wu H. Inflammasome-activated gasdermin D causes pyroptosis by forming membrane pores. Nature. (2016) 535:153–8. doi: 10.1038/nature18629 PMC5539988 27383986

[54] Thieblemont N Wright HL Edwards SW Witko-Sarsat V. Human neutrophils in auto-immunity. Semin Immunol. (2016) 28:159–73. doi: 10.1016/j.smim.2016.03.004 27036091

[55] Kim J Gupta R Blanco LP Yang S Shteinfer-Kuzmine A Wang K. VDAC oligomers form mitochondrial pores to release mtDNA fragments and promote lupus-like disease. Science. (2019) 366:1531–6. doi: 10.1126/science.aav4011 PMC8325171 31857488

[56] Miao N Wang Z Wang Q Xie H Yang N Wang Y. Oxidized mitochondrial DNA induces gasdermin D oligomerization in systemic lupus erythematosus. Nat Commun. (2023) 14:872. doi: 10.1038/s41467-023-36522-z 36797275 PMC9935630

[57] Jiang K Tu Z Chen K Xu Y Chen F Xu S. Gasdermin D inhibition confers antineutrophil-mediated cardioprotection in acute myocardial infarction. J Clin Invest. (2022) 132:e151268. doi: 10.1172/JCI151268 34752417 PMC8718151

[58] Fan X Han J Zhong L Zheng W Shao R Zhang Y. Macrophage-derived GSDMD plays an essential role in atherosclerosis and cross talk between macrophages via the mitochondria-STING-IRF3/NF-κB axis. Arterioscler Thromb Vasc Biol. (2024) 44:1365–78. doi: 10.1161/ATVBAHA.123.320612 38695170

[59] Yang CA Huang ST Chiang BL. Sex-dependent differential activation of NLRP3 and AIM2 inflammasomes in SLE macrophages. Rheumatol (Oxford). (2015) 54:324–31. doi: 10.1093/rheumatology/keu318 25161312

[60] Vlachakis PK Theofilis P Kachrimanidis I Giannakopoulos K Drakopoulou M Apostolos A. The role of inflammasomes in heart failure. Int J Mol Sci. (2024) 25:5372. doi: 10.3390/ijms25105372 38791409 PMC11121241

[61] Luo B Wang F Li B Dong Z Liu X Zhang C. Association of nucleotide-binding oligomerization domain-like receptor 3 inflammasome and adverse clinical outcomes in patients with idiopathic dilated cardiomyopathy. Clin Chem Lab Med. (2013) 51:1521–8. doi: 10.1515/cclm-2012-0600 23382313

[62] Wu X Yang J Wu J Yang X. Therapeutic potential of MCC950, a specific inhibitor of NLRP3 inflammasome in systemic lupus erythematosus. BioMed Pharmacother. (2024) 172:116261. doi: 10.1016/j.biopha.2024.116261 38340397

[63] Shi Y Zhao L Wang J Liu S Zhang Y Qin Q. The selective NLRP3 inflammasome inhibitor MCC950 improves isoproterenol-induced cardiac dysfunction by inhibiting cardiomyocyte senescence. Eur J Pharmacol. (2022) 937:175364. doi: 10.1016/j.ejphar.2022.175364 36336012

[64] Zeng W Wu D Sun Y Suo Y Yu Q Zeng M. The selective NLRP3 inhibitor MCC950 hinders atherosclerosis development by attenuating inflammation and pyroptosis in macrophages. Sci Rep. (2021) 11:19305. doi: 10.1038/s41598-021-98437-3 34588488 PMC8481539

[65] Abbate A Toldo S Marchetti C Kron J Van Tassell BW Dinarello CA. Interleukin-1 and the inflammasome as therapeutic targets in cardiovascular disease. Circ Res. (2020) 126:1260–80. doi: 10.1161/CIRCRESAHA.120.315937 PMC8760628 32324502

[66] Shen S Duan J Hu J Qi Y Kang L Wang K. Colchicine alleviates inflammation and improves diastolic dysfunction in heart failure rats with preserved ejection fraction. Eur J Pharmacol. (2022) 929:175126. doi: 10.1016/j.ejphar.2022.175126 35779623

[67] Lee KH Kronbichler A Park DD Park Y Moon H Kim H. Neutrophil extracellular traps (NETs) in autoimmune diseases: A comprehensive review. Autoimmun Rev. (2017) 16:1160–73. doi: 10.1016/j.autrev.2017.09.012 28899799

[68] Singh J Boettcher M Dölling M Heuer A Hohberger B Leppkes M. Moonlighting chromatin: when DNA escapes nuclear control. Cell Death Differ. (2023) 30:861–75. doi: 10.1038/s41418-023-01124-1 PMC9907214 36755071

[69] Liu ML Lyu X Werth VP. Recent progress in the mechanistic understanding of NET formation in neutrophils. FEBS J. (2022) 289:3954–66. doi: 10.1111/febs.16036 PMC9107956 34042290

[70] Goldberg MW Huttenlauch I Hutchison CJ Stick R. Filaments made from A- and B-type lamins differ in structure and organization. J Cell Sci. (2008) 121:215–25. doi: 10.1242/jcs.022020 18187453

[71] Li Y Li M Weigel B Mall M Werth VP Liu ML. Nuclear envelope rupture and NET formation is driven by PKCα-mediated lamin B disassembly. EMBO Rep. (2020) 21:e48779. doi: 10.15252/embr.201948779 32537912 PMC7403722

[72] Amulic B Knackstedt SL Abu Abed U Deigendesch N. Cell-cycle proteins control production of neutrophil extracellular traps. Dev Cell. (2017) 43:449–62.e5. doi: 10.1016/j.devcel.2017.10.013 29103955

[73] Pisareva E Mihalovičová L Pastor B Kudriavtsev A Mirandola A Mazard T. Neutrophil extracellular traps have auto-catabolic activity and produce mononucleosome-associated circulating DNA. Genome Med. (2022) 14:135. doi: 10.1186/s13073-022-01125-8 36443816 PMC9702877

[74] Li M Weng L Yu D Yang G Hao J. Increased formation of neutrophil extracellular traps induced by autophagy and identification of autophagy-related biomarkers in systemic lupus erythematosus. Exp Dermatol. (2024) 33:e14881. doi: 10.1111/exd.14881 37539924

[75] Dieker J Tel J Pieterse E Thielen A Rother N Bakker M. Circulating apoptotic microparticles in systemic lupus erythematosus patients drive the activation of dendritic cell subsets and prime neutrophils for NETosis. Arthritis Rheumatol. (2016) 68:462–72. doi: 10.1002/art.39417 26360137

[76] Rother N Pieterse E Lubbers J Hilbrands L van der Vlag J. Acetylated histones in apoptotic microparticles drive the formation of neutrophil extracellular traps in active lupus nephritis. Front Immunol. (2017) 8:1136. doi: 10.3389/fimmu.2017.01136 28959262 PMC5604071

[77] Lood C Blanco LP Purmalek MM Carmona-Rivera C De Ravin SS Smith CK. Neutrophil extracellular traps enriched in oxidized mitochondrial DNA are interferogenic and contribute to lupus-like disease. Nat Med. (2016) 22:146–53. doi: 10.1038/nm.4027 PMC4742415 26779811

[78] Fortner KA Blanco LP Buskiewicz I Huang N Gibson PC Cook DL. Targeting mitochondrial oxidative stress with MitoQ reduces NET formation and kidney disease in lupus-prone MRL-lpr mice. Lupus Sci Med. (2020) 7:e000387. doi: 10.1136/lupus-2020-000387 32343673 PMC7199895

[79] Henning S Reimers T Abdulahad W Fierro JJ Doornbos-van der Meer B Bootsma H. Low density granulocytes and neutrophil extracellular trap formation are increased in incomplete systemic lupus erythematosus. Rheumatol (Oxford). (2024) keae300. doi: 10.1093/rheumatology/keae300 PMC11879334 38775454

[80] Pertiwi KR van der Wal AC Pabittei DR Mackaaij C van Leeuwen MB Li X. Neutrophil extracellular traps participate in all different types of thrombotic and haemorrhagic complications of coronary atherosclerosis. Thromb Haemost. (2018) 118:1078–87. doi: 10.1055/s-0038-1641749 29672788

[81] Hofbauer TM Mangold A Scherz T Seidl V Panzenböck A Ondracek AS. Neutrophil extracellular traps and fibrocytes in ST-segment elevation myocardial infarction. Basic Res Cardiol. (2019) 114:33. doi: 10.1007/s00395-019-0740-3 31312919 PMC6647191

[82] Moore S Juo HH Nielsen CT Tyden H Bengtsson AA Lood C. Role of neutrophil extracellular traps regarding patients at risk of increased disease activity and cardiovascular comorbidity in systemic lupus erythematosus. J Rheumatol. (2020) 47:1652–60. doi: 10.3899/jrheum.190875 PMC7520909 31839592

[83] Nappi F Bellomo F Avtaar Singh SS. Worsening thrombotic complication of atherosclerotic plaques due to neutrophils extracellular traps: A systematic review. Biomedicines. (2023) 11:113. doi: 10.3390/biomedicines11010113 36672621 PMC9855935

[84] Ibrahim N Eilenberg W Neumayer C Brostjan C. Neutrophil extracellular traps in cardiovascular and aortic disease: A narrative review on molecular mechanisms and therapeutic targeting. Int J Mol Sci. (2024) 25:3983. doi: 10.3390/ijms25073983 38612791 PMC11012109

[85] Yao M Ma J Wu D Fang C Wang Z Guo T. Neutrophil extracellular traps mediate deep vein thrombosis: from mechanism to therapy. Front Immunol. (2023) 14:1198952. doi: 10.3389/fimmu.2023.1198952 37680629 PMC10482110

[86] Chrysanthopoulou A Gkaliagkousi E Lazaridis A Arelaki S Pateinakis P Ntinopoulou M. Angiotensin II triggers release of neutrophil extracellular traps, linking thromboinflammation with essential hypertension. JCI Insight. (2021) 6:e148668. doi: 10.1172/jci.insight.148668 34324440 PMC8492353

[87] Krishnan J Hennen EM Ao M Kirabo A Ahmad T de la Visitación N. NETosis drives blood pressure elevation and vascular dysfunction in hypertension. Circ Res. (2024) 134:1483–94. doi: 10.1161/CIRCRESAHA.123.323897 PMC11116040 38666386

[88] Knight JS Luo W O'Dell AA Yalavarthi S Zhao W Subramanian V. Peptidylarginine deiminase inhibition reduces vascular damage and modulates innate immune responses in murine models of atherosclerosis. Circ Res. (2014) 114:947–56. doi: 10.1161/CIRCRESAHA.114.303312 PMC4185401 24425713

[89] Lin W Chen H Chen X Guo C. The roles of neutrophil-derived myeloperoxidase (MPO) in diseases: the new progress. Antioxidants (Basel). (2024) 13:132. doi: 10.3390/antiox13010132 38275657 PMC10812636

[90] Chen W Tumanov S Kong SMY Cheng D Michaëlsson E Bongers A. Therapeutic inhibition of MPO stabilizes pre-existing high risk atherosclerotic plaque. Redox Biol. (2022) 58:102532. doi: 10.1016/j.redox.2022.102532 36375379 PMC9663534

[91] Yang K Gao R Chen H Hu J Zhang P Wei X. Myocardial reperfusion injury exacerbation due to ALDH2 deficiency is mediated by neutrophil extracellular traps and prevented by leukotriene C4 inhibition. Eur Heart J. (2024) 45:1662–80. doi: 10.1093/eurheartj/ehae205 PMC11089336 38666340

[92] Li S Huang Y. Ferroptosis: an iron-dependent cell death form linking metabolism, diseases, immune cell and targeted therapy. Clin Transl Oncol. (2022) 24:1–12. doi: 10.1007/s12094-021-02669-8 34160772 PMC8220428

[93] Yu Y Yan Y Niu F Wang Y Chen X Su G. Ferroptosis: a cell death connecting oxidative stress, inflammation and cardiovascular diseases. Cell Death Discov. (2021) 7:193. doi: 10.1038/s41420-021-00579-w 34312370 PMC8313570

[94] Zhang W Liu Y Liao Y Zhu C Zou Z. GPX4, ferroptosis, and diseases. Biomed Pharmacother. (2024) 174:116512. doi: 10.1016/j.biopha.2024.116512 38574617

[95] Li P Jiang M Li K Li H Zhou Y Xiao X. Glutathione peroxidase 4-regulated neutrophil ferroptosis induces systemic autoimmunity. Nat Immunol. (2021) 22:1107–17. doi: 10.1038/s41590-021-00993-3 PMC8609402 34385713

[96] Chen Q Xiang M Gao Z Lvu F Sun Z Wang Y. The role of B-cell ferroptosis in the pathogenesis of systemic lupus erythematosus. Clin Immunol. (2023) 256:109778. doi: 10.1016/j.clim.2023.109778 37730009

[97] Yang B Hou S Huang S Li H Li Y. Ferroptosis inhibitor regulates the disease progression of systematic lupus erythematosus mice model through Th1/Th2 ratio. Curr Mol Med. (2023) 23:799–807. doi: 10.2174/1566524022666220525144630 35619279

[98] Zhang Z Yang Z Wang S Wang X Mao J. Decoding ferroptosis: Revealing the hidden assassin behind cardiovascular diseases. BioMed Pharmacother. (2024) 176:116761. doi: 10.1016/j.biopha.2024.116761 38788596

[99] Skaggs BJ Hahn BH McMahon M. Accelerated atherosclerosis in patients with SLE–mechanisms and management. Nat Rev Rheumatol. (2012) 8:214–23. doi: 10.1038/nrrheum.2012.14 PMC3765069 22331061

[100] Bai T Li M Liu Y Qiao Z Wang Z. Inhibition of ferroptosis alleviates atherosclerosis through attenuating lipid peroxidation and endothelial dysfunction in mouse aortic endothelial cell. Free Radic Biol Med. (2020) 160:92–102. doi: 10.1016/j.freeradbiomed.2020.07.026 32768568

[101] Zhou Y Zhou H Hua L Hou C Jia Q Chen J. Verification of ferroptosis and pyroptosis and identification of PTGS2 as the hub gene in human coronary artery atherosclerosis. Free Radic Biol Med. (2021) 171:55–68. doi: 10.1016/j.freeradbiomed.2021.05.009 33974977

[102] Tian H Xiong Y Zhang Y Leng Y Tao J Li L. Activation of NRF2/FPN1 pathway attenuates myocardial ischemia-reperfusion injury in diabetic rats by regulating iron homeostasis and ferroptosis. Cell Stress Chaperones. (2021) 27:149–64. doi: 10.1007/s12192-022-01257-1 PMC8943074 35124772

[103] Chen HY Xiao ZZ Ling X Xu RN Zhu P Zheng SY. ELAVL1 is transcriptionally activated by FOXC1 and promotes ferroptosis in myocardial ischemia/reperfusion injury by regulating autophagy. Mol Med. (2021) 27:14. doi: 10.1186/s10020-021-00271-w 33568052 PMC7874472

[104] Feng Y Madungwe NB Imam Aliagan AD Tombo N Bopassa JC. Liproxstatin-1 protects the mouse myocardium against ischemia/reperfusion injury by decreasing VDAC1 levels and restoring GPX4 levels. Biochem Biophys Res Commun. (2019) 520:606–11. doi: 10.1016/j.bbrc.2019.10.006 PMC7457545 31623831

